# Evaluating an Implementation Strategy Bundle in Title X‐Funded Safety‐Net Family Planning Clinics to Increase HIV Testing and PrEP Access Among Cis‐Gender Women in Atlanta, United States: Lessons Learned From Provider Focus Groups

**DOI:** 10.1002/jia2.70212

**Published:** 2026-10-08

**Authors:** Jessica M. Sales, Katherine M. Anderson, Deja Er, Peyton Williams, Sara Sullivan, Michael W. Brooks, Melissa Kottke, Anandi Sheth

**Affiliations:** ^1^ Department of Behavioral, Social, and Health Education Sciences, Rollins School of Public Health Emory University Atlanta Georgia USA; ^2^ Division of Infectious Diseases, Department of Medicine Emory University School of Medicine Atlanta Georgia USA; ^3^ The Family Health Centers of Georgia, Inc., Georgia Family Planning System Atlanta Georgia USA; ^4^ Department of Gynecology and Obstetrics, Jane Fonda Center Emory University School of Medicine Atlanta Atlanta Georgia USA

**Keywords:** family planning, HIV prevention, implementation strategies, pre‐exposure prophylaxis, Title X, women's health

## Abstract

**Introduction:**

Pre‐exposure prophylaxis (PrEP) remains underutilized by women in the United States, in part because PrEP is not widely available in women's healthcare settings. We developed a bundle of tailored implementation strategies (trainings, technical assistance [TA], resources) to support HIV prevention delivery in Title X‐funded family planning clinics across Atlanta, Georgia (GA). Guided by the interactive systems framework, we partnered with GA's largest Title X grant holder to provide these strategies to this network. We conducted an evaluation of clinic strategy use and described pre‐ and post‐strategy engagement HIV prevention/PrEP practices.

**Methods:**

Using a qualitative descriptive design, between January 2022 and November 2023, 47 staff, providers and leadership from 11 clinical groups representing 22 of 31 Atlanta Title X clinics (71%) separately participated in baseline (pre‐strategy) focus groups (FGs); 10 groups participated in FGs 1‐year post‐strategy(s). Rapid qualitative analysis of FGs was performed. Chart‐abstracted measures were used to describe clinical group‐aggregate HIV testing numbers 1 year prior to and 1 year after implementation strategy engagement.

**Results:**

Twenty‐two trainings were provided (*n* = 358 participants), with two TA and 53 requests received across 11 clinical groups. Clinics reported training and resource implementation strategies were acceptable and helped increase their readiness/capacity to provide PrEP, but scheduling trainings was sometimes challenging. Due to significant staff turnover, many noted that refresher trainings were or would be beneficial. TA was underutilized despite its potential need by clinical groups. At baseline, few clinics (<5; 22%) were providing PrEP. At follow‐up, eight clinical groups were offering PrEP; one offered referrals to a PrEP‐specific clinic. Three groups were able to provide injectable PrEP; two groups were not offering PrEP consistently or at all. Most groups (8 of 11; 73%) reported increases in total HIV tests in the 12 months after strategy engagement.

**Conclusions:**

Implementation strategies were utilized by clinics. Tailored trainings and resources were perceived as helpful for improving HIV testing, and PrEP provision in family planning settings and sustainable access to them via a centralized agency may be necessary to overcome ongoing capacity‐related barriers. Additional strategies are necessary to ensure clinics are aware of and feel comfortable accessing the implementation strategies over time.

## 1 Introduction

Although new HIV diagnoses in the United States (U.S.) are trending down in recent years, HIV incidence rates in cisgender women continue to remain stagnant, accounting for about one in five of the approximately 37,600 new HIV acquisitions in 2022 [1, 2]. More than half of new acquisitions occur in the South [3], and Georgia (GA) leads the U.S. in rates of new HIV acquisitions, with the majority of cases concentrated in the Atlanta metropolitan area [3]. Fulton and DeKalb counties report rates of 46.5 and 60.6 per 100,000, respectively [3]. Reducing HIV among women in GA remains a public health priority, and targeted strategies are needed to reach cisgender women and achieve an end to HIV.

HIV pre‐exposure prophylaxis (PrEP) is safe and effective at preventing HIV in cisgender women [4–6]. More choices are becoming available with advances in biomedical HIV prevention methods. This has led to the emergence of three effective PrEP options approved for use by cisgender women, including a daily oral PrEP, bimonthly injectable PrEP administered in a clinical setting and twice‐yearly injectable PrEP administered by healthcare providers [4, 6, 7, 8]. Yet, cisgender women still account for less than 10% of PrEP users [3], and cisgender women's unmet need for PrEP was more than double men's unmet need, in part because PrEP is not widely available in women's healthcare settings [9, 10]. Anchoring PrEP care in family planning services that are trusted, routinely accessed and perceived as beneficial by patients may play a critical role in advancing the goals of the Ending the HIV Epidemic (EHE) initiative and reducing U.S. cisgender women's HIV rates [2, 3, 4, 5].

The federally funded Title X National Family Planning Program supports a nationwide network of ∼4000 family planning sites with nearly three million clients annually, 87% of whom are female [11] (Note: Title X requires reporting patients’ sex rather than gender). The programme is designed to ensure contraception access, particularly for low‐income individuals, but also funds preventive services including HIV testing and prevention. Further, the body overseeing the Title X programme—the Office of Population Affairs—recommends use of evidence‐based preventative screening and counselling, as well as HIV prevention service [12]. While most Title X clients are cisgender women, clinics also serve men and, therefore, are poised to play an essential role in expanding access for all populations. In GA, the largest Title X grant is operated by Georgia Family Planning System (GFPS) via a statewide network of ∼180 Federally Qualified Health Centers. In 2023, this network of clinics received Title X funding to provide subsidized family planning and preventative health services such as HIV and sexually transmitted infections (STI) testing to more than 124,000 cisgender women [11], including 31 clinics in the four metro Atlanta high‐priority EHE counties.

The integration of universal HIV testing into Title X clinics helps normalize HIV testing and prevention counselling, increases testing acceptability, improves health outcomes and is a key component of PrEP care [13, 14, 15]. The U.S. Centers for Disease Control and Prevention (CDC) recommends routine, universal, opt‐out HIV testing for persons ages 13–64 years [16]. Yet, in collaboration with GFPS, we found that only 20% of GA Title X clinics used all elements of CDC HIV testing recommendations, and 40% used none [17]. The underutilization of HIV testing can result in missed opportunities for HIV prevention counselling. PrEP delivery in Title X clinics in the Southern United States, including GA, also remains low, despite clinical guidelines having incorporated PrEP. Sales et al. surveyed nearly 600 providers/staff working in 286 Title X clinics across the South; only 22% of clinics provided any PrEP services (including through referral), and the Southeastern region (including Atlanta) had the fewest clinics offering PrEP [9].

Based on formative findings from a prior needs assessment we conducted with Title X clinics in metro Atlanta [18, 19, 20], we developed a bundle of tailored implementation strategies (trainings, technical assistance [TA], resources) to support increased implementation of HIV prevention practices including HIV testing and PrEP delivery in Title X clinics across Atlanta (see Table 1). From prior research and our formative work, we also learned that these strategies should be sustainably available given the changing Title X landscape and high staff turnover within clinics, requiring a systems‐level approach. Thus, guided by the interactive systems framework (ISF) [21], we partnered with GFPS to provide these strategies on an ongoing basis to their clinical network. The ISF is a system‐level model to help coordinate communication and work across agencies working in collaboration to sustainably improve implementation and reach of new HIV prevention practices. The ISF consists of three systems: “Prevention Delivery” (implements the innovation in the real world), “Prevention Support” (provides ongoing training and TA to users in the field) and “Prevention Synthesis and Translation” (conducts research and distils information about innovations in user‐friendly formats (see Figure 1). ISF has been used successfully by the CDC in their multi‐site capacity‐building effort to reduce teen pregnancies in the United States [22], but its use to guide systems‐level HIV prevention improvements in the family planning safety‐net network and evaluations of its utility are non‐existent.

**FIGURE 1:**
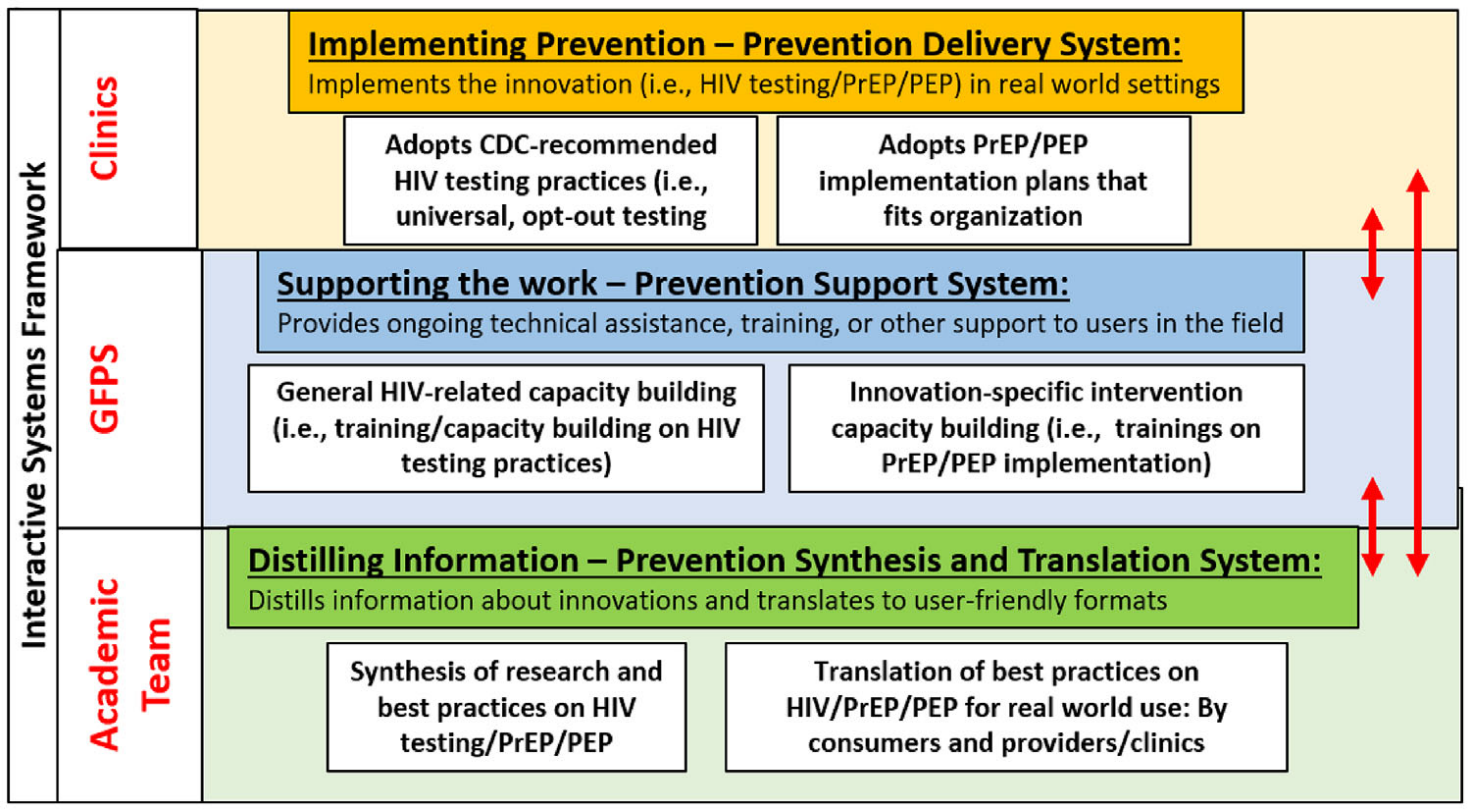
Interactive systems framework approach to supporting Title X clinics implementation of HIV prevention services in metro Atlanta. Abbreviations: CDC, Centers for Disease Control and Prevention; GFPS, Georgia Family Planning System; PEP, post‐exposure prophylaxis; PrEP, pre‐exposure prophylaxis.

After preparing the tailored bundle of implementation strategies to support HIV prevention practice implementation in the Title X clinical network across Atlanta, we conducted a qualitative descriptive evaluation of clinics use of the strategies and explored the perceived impact of the strategies on HIV prevention practices, namely HIV testing and PrEP implementation within their clinics. Evaluation activities included monitoring Title X clinic use of the implementation strategies offered by GFPS and exploring their perception of the strategies offered by GFPS on HIV testing and PrEP implementation, with a particular focus on reach among cisgender women in their clinics. Based on the ISF, we presumed that clinics would utilize the strategies, describe them as acceptable and appropriate, and utilization of implementation strategies would be perceived as supporting clinics’ HIV testing and PrEP implementation over time. Findings from this study can inform future scaling of the implementation strategy bundle across GA, as well as demonstrate the feasibility and utility of employing an ISF approach for improving HIV prevention practices within the Title X National Family Planning Program and other healthcare systems more broadly.

## 2 Methods

### 2.1 Setting

In the city of Atlanta, GA, the rate of new HIV acquisitions (44 per 100,000) is higher than the rate in GA (28 per 100,000), the U.S. Southern region (19 per 100,000) and the United States as a whole (14 per 100,000) [23]. The four counties comprising the Atlanta metro area (Cobb, DeKalb, Fulton and Gwinnett) are each identified in the *EHE* Initiative as priority counties [24], indicating high need for prevention efforts. Within these four counties, at the time of study initiation, there were 31 Title X clinics, representing approximately 20% of Title X clinics in GA [25].

### 2.2 Procedures

This work was conducted as part of a larger study to develop, implement and evaluate tailored implementation strategies to support HIV prevention practices implementation at metro Atlanta Title X clinics. Specifically, a bundle of implementation strategies, including trainings, TA and patient and provider‐facing resources, were offered to Title X clinical groups located in metro Atlanta (see Table 1). Clinical group refers to a group of clinics all operated by the same agency. Clinic refers to individual clinical sites. All Title X‐funded clinical groups located in metro Atlanta were eligible to receive the implementation strategies. Eligible clinical groups who were interested in potentially receiving training, TA or other resources were invited to participate in a baseline focus group (FG) with the study team. Clinical groups were asked to invite members of their staff who were part of HIV prevention service delivery (e.g. navigators, medical assistants, providers) and/or clinical decision‐making (e.g. medical director) to participate in FGs. After FG participation, clinical groups could request and receive any number of implementation strategies from the menu, tailoring strategy use to their needs. Training, TA and resource requests were documented. One year after onset of strategy engagement, clinical groups were invited to participate in a follow‐up FG to descriptively evaluate the trainings and other strategy uses’ impact on HIV prevention services, as well as to identify additional strategies to support HIV testing and PrEP services. Chart‐abstracted measures were used to describe clinical group aggregate HIV testing numbers 1 year prior to and 1 year after implementation strategy engagement.

**TABLE 1:** Menu of PrEP‐related trainings, technical assistance and resources offered to Title X clinics.

Training title	Training description
Overview of HIV testing and PrEP	General overview of PrEP/PEP (what it is, how it works, potential benefits); tips for conversations about PrEP; overview of HIV testing (universal, opt‐out, routine) geared towards non‐clinical staff; 30 min
PrEP care for clinicians	Steps for PrEP implementation including interpreting HIV tests and labs, medication dosing and prescribing PrEP, monitoring and follow‐up, and women's considerations; 60 min
PrEP insurance navigation	How to navigate insurances/medication assistance programmes for PrEP
Long‐acting injectable PrEP	Overview of new PrEP medication options and addressing barriers to implementation
Alternative PrEP delivery	Discuss PrEP care models (i.e. same‐day and telehealth PrEP)
Taking a sexual history	Improving skills in taking a detailed, non‐judgemental sexual history
STIs (2021 guidelines)	Review CDC 2021 STI diagnosis and management guidelines
**Technical assistance**	
Follow‐in support for any PrEP trainings	Individualized, tailored follow‐up and hands‐on support specific to any of the trainings offered
PrEP implementation planning consult visit	To facilitate the development of a plan for PrEP implementation in their clinic
PrEP and EHR consultation	To provide technical assistance on how to incorporate PrEP care steps into the EHR and/or utilize the EHR to track PrEP metrics
Consultation on alternative PrEP delivery	To assist clinics that want to incorporate novel PrEP delivery models (e.g. same‐day PrEP and telehealth PrEP)
**PrEP resource materials**	
Patient‐facing PrEP materials	Flyers, handouts, posters including with specific content for women (available in multiple languages)
Provider‐facing materials	Reference sheets on HIV testing, PrEP counselling, and/or PrEP steps, and sexual health assessment
Sexual health assessments	Standardized sexual health assessments that can help identify patients who may most benefit from PrEP, inclusive of unique considerations for women
Optout HIV testing guidance	Template example of opt‐out of HIV testing consents

Abbreviations: EHR, electronic health record; PEP, post‐exposure prophylaxis; PrEP, pre‐exposure prophylaxis.

#### 2.2.1 Baseline FGs

Between January 2022 and November 2023, 47 staff, providers and clinic leadership from 11 Title X clinical groups representing 22 of 31 Atlanta Title X clinics (71%) participated in 11 separate baseline (pre‐training) FGs (one per clinical group). Based on the content of the FGs, clinical groups could identify the number of clinic leadership and staff they felt should be invited to participate to provide comprehensive information for their clinical group. Thus, clinics determined the number of individuals that would be best suited to speak to their sites’ HIV prevention services. The FGs aimed to gather information on current sexual and reproductive healthcare services, including sexual history taking, HIV counselling and testing, PrEP care processes, and to assess the acceptability and interest in PrEP capacity‐building activities specifically tailored to meet individual clinical groups’ needs. FG participants were presented with a menu of trainings, TA, and patient‐ and provider‐facing resources (Table 1) developed by the research team and designed to support implementation of HIV prevention in Title X clinics. FGs were conducted in‐person or via Zoom Videoconferencing and lasted an average of 38 min. A note‐taker was present at each FG; discussions were recorded and then sent to an external transcription service. Staff members checked transcriptions against the original recordings and removed identifying information. More details pertaining to the baseline FG results, including how they were used to tailor the implementation bundle, are reported elsewhere [26].

#### 2.2.2 Implementation Strategy Engagement

Clinical groups were offered a menu of nine 30–60 min in‐person or virtual trainings, TA options and resources (Table 1). Trainings were tailored for cisgender women and delivered by infectious disease physicians with experience delivering HIV prevention and PrEP care services and covered guideline‐based care.

#### 2.2.3 One‐Year Follow‐Up FGs

Between March 2023 and June 2024, 41 staff, providers and clinic leadership from 10 of the 11 clinical groups participated in separate 1‐year follow‐ups. In some instances, follow‐up FG participants were the same as those who participated in baseline. However, there was significant turnover at all levels of clinical group staffing, resulting in new participants over time. FGs followed the same format as baseline FGs, but focused on gathering information on changes in HIV prevention services since strategy engagement and their assessment of the bundle of implementation strategies, including recommendations for additional implementation strategies.

#### 2.2.4 Chart Abstracted HIV Testing Data

Chart‐abstracted measures were reported by clinical groups to GFPS pertaining to their clinical group‐level aggregate HIV testing pre‐ and post‐strategy engagement. The number of HIV tests performed within the scope of the Title X services was collected and reported to GFPS quarterly by each clinical group. These data are already routinely collected for reporting requirements by the Health Resources and Services Administration, Office of Population Affairs for all Title X clinical sites via the Family Planning Annual Report (FPAR). Site‐specific, quarterly aggregate data including the number of HIV tests performed for male and female family planning users from calendar years 2021–2024 were extracted by all 11 clinical groups for tabulation and analysis using descriptive statistics.

#### 2.2.5 Ethical Oversight

All FG participants worked at Title X clinics in metro Atlanta and provided verbal consent to participate. Verbal consent was documented by the study staff member conducting the informed consent and facilitating the FG. Participants each received a $50 gift card for their participation. All procedures were approved by the Institutional Review Board at Emory University (STUDY00002950).

### 2.3 Analysis

#### 2.3.1 FG Analysis

Baseline and follow‐up FGs were analysed using rapid analytic methods, based on a modified RADar technique [27], as previously described [26]. For each set of FGs, graduate‐level researchers with training in qualitative methods created a rapid analysis matrix for each time point, with each row indicating an FG ID, and each column indicating a question in the FG guide. One researcher populated the matrices with de‐identified direct quotes from the transcribed FGs; a second researcher reviewed the matrices, narrowing quotes to directly applicable content. For each set of FGs, a narrative summary was developed based on the salience of topics across FGs. Each of these narratives was iteratively reviewed and revised by the larger study team to increase clarity and relevance of the findings for evaluation. To compare results across time points, the analyst who authored the summaries developed a table where each row represented a domain, and columns represented the baseline and follow‐up time points. The analyst then reviewed domain‐specific results across time points and developed a summary of differences. These results were developed into a narrative with exemplar quotes, which was reviewed by the larger study team.

#### 2.3.2 Chart‐Abstracted HIV Testing Analysis

The total number of HIV tests performed each quarter from 2021 through 2024 was reported at the aggregate level for each clinical group, with testing numbers also stratified by patient sex (male/female). Total number of HIV tests and HIV tests among female patients only performed by each clinical group per quarter for the 12 months preceding participation in a training and for the 12 months after training were summed to create a pre‐training and post‐training value for each clinical group (total, female only). Percent change scores from pre‐training to post‐training for total HIV tests and HIV tests among female patients only were calculated per group.

## 3 Results

### 3.1 Participants

Baseline and follow‐up FGs had four participants on average. Participants included health educators/patient navigators/programme coordinators (26%), physicians (21%), advanced practice providers (APPs) (13%), nurses (13%), medical assistants (15%) and clinic administrators (12%).

### 3.2 Implementation Strategy Engagement

Between April 2022 and June 2024, 22 trainings were provided to the 11 clinical groups (*n* = 358 participants). Eight were “Overview of PrEP” trainings; participants were medical assistants (31%), nurses (12%), APPs (6%) and other (37%). Twelve were “PrEP Care for Clinicians” trainings; participants were mostly APPs (20%), physicians (17%), nurses (15%) and other (37%). One sexual history taking and one long‐acting injectable PrEP training were delivered. Two TA requests and 53 requests for resources were made across groups. Table 2 provides details of each clinical group's strategy engagement between January 2022 and June 2024.

**TABLE 2:** Implementation strategy engagement, by strategy type and date of engagement for each clinical group.

CLINICAL GROUP	STRATEGY ENGAGEMENT 1: TRAININGS	STRATEGY ENGAGEMENT 2: PREP RESOURCES	STRATEGY ENGAGEMENT 3: TECHNICAL ASSISTANCE
1	PrEP care for clinicians (4/2022) Overview of HIV testing and PrEP (5/2022) Taking a sexual history (1/2023)	Patient‐facing PrEP materials (2/2022, 04/2022, 1/2023) Provider‐facing materials (04/2022, 9/2022, 1/2023)	PrEP implementation planning consult visit (5/2022)
2	Long‐acting injectable PrEP (11/2023)	Provider‐facing materials (11/2023)	
3	Overview of HIV testing and PrEP (6/2022) PrEP care for clinicians (4/2024)	Patient‐facing PrEP materials (2/2022, 9/2022, 3/2023) Provider‐facing materials (2/2022, 3/2023)	
4	Overview of HIV testing and PrEP (4/2022) PrEP care for clinicians (5/2022) PrEP care for clinicians (6/2024)	Patient‐facing PrEP materials (1/2022, 5/2022, 6/2024) Provider‐facing materials (5/2022)	PrEP training follow‐in support (11/2022)
5	PrEP care for clinicians (6/2022) Overview of HIV testing and PrEP (9/2022)	Patient‐facing PrEP materials (2/2022, 6/2022, 5/2023) Provider‐facing materials (6/2022, 5/2023)	
6	Overview of HIV testing and PrEP (9/2022) PrEP care for clinicians (9/2022) PrEP care for clinicians (3/2023) Overview of HIV testing and PrEP (11/2023)	Patient‐facing PrEP materials (7/2022, 8/2022, 10/2022, 3/2023, 11/2023) Provider‐facing materials (7/2022, 8/2022, 10/2022, 3/2023)	
7	PrEP care for clinicians (2/2023)	Patient‐facing PrEP materials (3/2023) Provider‐facing materials (3/2023)	
8	PrEP care for clinicians (2/2023) PrEP care for clinicians (11/2023)	Patient‐facing PrEP materials (1/2023, 2/2023, 11/2023) Provider‐facing materials (2/2023, 11/2023)	
9	Overview of HIV testing and PrEP (9/2022) PrEP care for clinicians (3/2024)	Patient‐facing PrEP materials (5/2022, 9/2022, 1/2024, 3/2024) Provider‐facing materials (9/2022, 1/2024)	
10	PrEP care for clinicians (11/2023)	Patient‐facing PrEP materials (5/2022, 11/2023) Provider‐facing materials (5/2022, 11/2023)	
11	Overview of HIV testing and PrEP (5/2022)	Patient‐facing PrEP materials (3/2022, 3/2023) Provider‐facing materials (3/2022, 5/2022)	

Abbreviation: PrEP, pre‐exposure prophylaxis.

### 3.3 Changes in HIV Testing and PrEP Services From Baseline to 1 Year Follow‐Up

At baseline, only two clinical groups provided opt‐out HIV testing, with an additional seven clinical groups indicating that they conduct “informal” opt‐out counselling (*n* = 2) or attempted to offer testing to most or all clients. At 1‐year follow‐up, most clinical groups (*n* = 7, 70%) indicated that clients are tested for HIV at least once in their time attending the clinic, or that they conduct universal opt‐out testing. At baseline, few individual clinic sites (<5 clinic sites across the 11 clinical groups with 22 total clinic sites combined; 22%) were providing PrEP. At 1‐year follow‐up, eight clinical groups were offering PrEP, while one clinical group offered referrals to a specific clinic in their group that provided PrEP. Of these clinical groups, three provided injectable PrEP in‐house or via referral; one clinic group was not offering PrEP regularly, while another group was not able to offer PrEP at all (see Table 3).

**TABLE 3:** HIV tests completed pre‐ and post‐strategy engagement by Title X family planning service site agencies.

Title X agency	Number of trainings	Number trained	Number resource/TA requests	Resources/TA provided	Tests 12 months pre‐training window	Tests 12 months post‐training window	Percent change
Clinic group 1^b^	3	51	7	Tailored PrEP implementation plan Patient‐facing PrEP education materials Provider PrEP reference sheet and resource guide	51	395	675%
Clinic group 2^a^,^b^	1	3	1	Provider long‐acting injectable PrEP resource guide	2212	1440	−35%
Clinic group 3^a^,^b^	2	28	5	Patient‐facing PrEP education materials Provider PrEP reference sheet and resource guide	2592	5051	95%
Clinic group 4^a^,^b^	3	70	5	Patient‐facing PrEP education materials Provider PrEP reference sheet and resource guide PrEP training follow‐in support	1682	1896	13%
Clinic group 5^b^	2	63	5	Patient‐facing PrEP education materials Provider PrEP reference sheet and resource guide	1736	2011	16%
Clinic group 6^a^,^b^	4	59	9	Patient‐facing PrEP education materials Provider PrEP reference sheet and resource guide	849	1070	26%
Clinic group 7	1	20	2	Patient‐facing PrEP education materials Provider PrEP reference sheet and resource guide	Not reported	440	N/A
Clinic group 8^b^	2	38	5	Patient‐facing PrEP education materials Provider PrEP reference sheet and resource guide	1388	1596	15%
Clinic group 9^b^	2	8	6	Patient‐facing PrEP education materials Provider PrEP reference sheet and resource guide	3293	2758	−16%
Clinic group 10^a^,^b^	1	4	4	Patient‐facing PrEP education materials Provider PrEP reference sheet and resource guide	6	535	8817%
Clinic group 11^a^	1	14	4	Patient‐facing PrEP education materials Provider PrEP reference sheet and resource guide	35	120	243%

*Note*: Percent change values marked “N/A” indicate division by zero (no tests reported during the pre‐training period).

Abbreviations: PrEP, pre‐exposure prophylaxis; TA, technical assistance.

^a^Providing PrEP at baseline.

^b^Providing PrEP at 1‐year follow‐up.

### 3.4 FG Findings From Baseline to Follow‐Up

Summaries of major themes describing the perceived utility of the implementation strategy bundle, and recommendations for additional implementation strategies to further support HIV prevention services in these clinical sites are presented below.

At baseline, participants discussed the need for multiple trainings on HIV prevention and PrEP and expressed interest in specific trainings on alternative PrEP delivery and taking a sexual history. At 1‐year follow‐up, all 11 clinical groups requested and received at least one training, with observed variability in number and type of training(s), and in requests for repeat training between clinical groups (Table 2). At follow‐up, clinics reported that the trainings received had generally been helpful in increasing their readiness and capacity to provide PrEP, with four clinics specifying that there was increased awareness and valuation of PrEP after training, as illustrated by one participant: “*So I think it got all the staff members on board, and then, of course, the clinician training was wonderful. It was really a good training for all of us*.” (Clinical group 1, Follow‐up). A participant at a separate clinic noted that at baseline, providers “*were not real comfortable [with PrEP]… so that training really helped,” and that before training began, “most of the staff, clinical and non‐clinical…They had no clue [about PrEP]*” (Clinical group 5, Follow‐up). While no trainings were identified as unhelpful, particularly beneficial topics included sexual history taking and creation of safe spaces/cultural sensitivity, which participants said taught them how to “*create a relationship where you feel you can go even further, and actually get the truth about what's going on with the patients, and what their perspectives are*” (Clinical group 3, Follow‐up). Participants also liked the training overviewing PrEP, for “giving us the birds‐eye view of what we are looking at, and then going into drilling down into the basics for the clinicians. I think that definitely helped” (Clinical group 5, Follow‐up).

In light of significant staff turnover reported by the majority of clinical groups, five clinical groups noted that repeated or “refresher” trainings were or would be beneficial. Second, participants noted that PrEP counselling occurred based on providers’ assessment of the patient's HIV risk or their comfort with PrEP counselling. As one participant said, “*It's generally when patients are coming in for a visit for something else… and they kind of meet that criteria for being high‐risk. Then there's a discussion that takes place around what is PrEP*” (Clinical group 11, Follow‐up). Noting the confluence of staffing changes and provider comfort with counselling, another participant stated, “*… in the future, we would like for more trainings. We do have new providers on board, so that'll definitely help. And it all depends on the comfort level of the providers, too…*” (Clinical group 5, Follow‐up).

Despite overwhelming acceptability of the trainings, a few challenges were noted for participating in trainings. Scheduling trainings, especially multiple trainings, was a universal challenge. Offering trainings during monthly staff meetings was reported by several clinical groups as a “great venue.” Most clinical groups had multiple locations and shared providers across sites, so they preferred a single training across all sites in a virtual setting, which was reported as a “good delivery model.” Yet, others reported lack of engagement by staff during virtual trainings; “*I like to think in person is always good…to ask questions directly face‐to‐face. I think we get Zoom fatigue, especially after three years of COVID and virtual meetings. So it's good to be in person*” (Clinical group 7, Follow‐up).

All clinical groups requested and received patient and/or provider‐facing resources (Tables 2 and 3) and were universally satisfied with the materials they received. Despite perceived success in increasing PrEP implementation following strategy engagement, clinics commonly cited limited uptake due to lack of patient interest and awareness, as exemplified by one participant: “*I feel like we have a really good process in place, it's just the reluctancy of the patient to agree to take the PrEP is kinda the problem, where we have a struggle*” (Clinical group 9, Follow‐up). While two clinical groups noted efforts to get the word out about PrEP, six clinical groups identified this as an area where additional support or resources would be beneficial, including more patient education materials and stigma reduction materials, with one clinic stating, “*I don't feel like we have any barriers as a health system, because I feel like we're giving the education, we're putting the materials out there.  We're laying it in their lap, it's just whether or not if they're receptive to it or not. …I think a lot of it comes with stigma*.” (Clinical group 9, Follow‐up).  One participant recommended alternative delivery options for patient education, stating, “*I personally feel like flyer handouts are—they're yesterday. [Laughter] I don't—every time we give people, it ends up on the floor in the trash…Why do that when I can just simply have them hit a QR code*” (Clinical group 3, Follow‐up).  One clinical group shared that materials to support providers in HIV prevention counselling would also be beneficial to support providers with educating and offering PrEP.

Despite offering TA as part of the implementation strategy bundle, very few clinical groups requested TA (Table 2). However, when asked about recommendations for additional ways that GFPS could support their HIV prevention services, several reported that lack of workflows, sexual health assessments in electronic medical records (EMRs) and patient insurance were ongoing challenges, all of which were existing trainings or TA offered in the bundle. Clinics offering injectable PrEP, in particular, faced substantial challenges navigating insurance for patients, yet none requested insurance navigation training or TA related to these challenges. Other barriers to implementation at follow‐up were time availability, patient citizenship/documentation status and lack of pharmacy. Clinic members also discussed possible additional implementation strategies to ameliorate several of these barriers, including identification of staff members to assist with PrEP funding navigation, training of non‐physician staff on counselling, establishment of protocols, collaboration with pharmacists and EMR integration. Again, with the exception of developing collaborations with pharmacists, the implementation strategies offered, particularly PrEP implementation planning TA, would meet many of these needs. One clinical group recommended that having outcome data relating to their efforts to increase HIV testing and PrEP would support their continued work by underscoring both need and effectiveness in their client population.

### 3.5 Chart‐Abstracted HIV Testing Numbers

Most clinical groups (8 of 11; 73%) reported an increase in total HIV tests in the 12 months after participating in training (Table 3), as well as an increase in HIV tests among female patients only (8 of 11; 73%; Table 4). Percent change in total HIV tests from the 12 months prior to training and the 12 months after training ranged from −35% to 8817% (Table 3); percent change in HIV tests among female patients only ranged from −45% to 682% (Table 4). There was no discernible relationship between the number of trainings, TA or resources a clinical group received and HIV testing numbers (total or for females only) (Table 3). Across all 11 clinical groups, there was an increase in HIV tests (total and females only) from 2021 levels, with increases observed from 2022 through 2024 (Table 5).

**TABLE 4:** Pre‐ and post‐training HIV tests completed by female family planning patients across metro Atlanta Title X‐funded clinic groups.

Title X agency	Number of trainings	Tests (12 months pre‐training)	Tests (12 months post‐training)	Percent change
Clinic group 1	3	34	266	682%
Clinic group 2	1	1087	602	−45%
Clinic group 3	2	2256	2170	−4%
Clinic group 4	3	357	654	83%
Clinic group 5	2	1285	1531	19%
Clinic group 6	4	843	1059	26%
Clinic group 7	1	0	307	N/A
Clinic group 8	2	1093	1290	18%
Clinic group 9	2	2016	2190	9%
Clinic group 10	1	0	307	N/A
Clinic group 11	1	28	77	175%

*Note*: Percent change values marked “N/A” indicate division by zero (no tests reported during the pre‐training period).

**TABLE 5:** Number of HIV tests performed by quarter by participating clinics, 2021−2024.

Confidential HIV tests	2021				2022				2023				2024			
	Q1	Q2	Q3	Q4	Q1	Q2	Q3	Q4	Q1	Q2	Q3	Q4	Q1	Q2	Q3	Q4
Female users	1802	1851	2099	1121	2052	2633	2372	2202	2563	2752	3032	4274	4224	3061	2880	1686
Male users	650	637	622	966	1004	1103	811	1102	1030	1047	1252	1494	1454	1555	1054	1146
All users	2452	2488	2721	2087	3056	3736	3183	3304	3593	3799	4284	5768	5678	4616	3934	2832
Total by year	9748				13,279				17,444				17,060			

## 4 Discussion

PrEP remains underutilized by women in the United States, in part because PrEP is not widely available in trusted women's healthcare settings. To address this gap, we developed and evaluated a bundle of tailored implementation strategies (trainings, TA, resources) to support increased HIV testing and PrEP delivery in Title X‐funded family planning clinics across Atlanta, GA—a high‐priority jurisdiction for the EHE initiative. Our evaluation study found that more clinical groups were offering HIV testing and PrEP after receiving training, clinical groups self‐reported the implementation strategy bundle helped increase their readiness and capacity to provide HIV prevention, and most clinical groups had chart‐reported increases in total HIV tests in the 12 months after participating in training.

Our findings are aligned with prior research demonstrating the value of provider training in the context of improving HIV testing and PrEP care. Among primary care providers, the Department of Infectious Diseases, Public Health Services of Amsterdam, implemented an educational intervention to improve HIV testing by general practitioners from 2015 to 2020, and observed a 7% increase in testing in an evaluation using laboratory data [28]. Specific to PrEP, several studies have found that after receiving training on PrEP, which resulted in increased knowledge of PrEP, providers began discussing PrEP with their patients more frequently as well as increased their PrEP prescriptions to patients [29, 30, 31, 32], though the majority of these studies focused on primary care settings. Among the limited evaluations of HIV testing and PrEP training in women's health settings, findings have been similarly promising. One pilot study of PrEP training implemented in four family planning clinics in Atlanta found that from pre‐training to post‐training, provider PrEP knowledge and confidence to identify women who may benefit from PrEP significantly increased, and the majority of patients surveyed across clinics reported receiving HIV prevention counselling and PrEP education during their visit after PrEP training [33]. Another study reported significantly increased PrEP prescriptions in one sexual health clinic in Washington, D.C., after providing educational trainings to medical providers, EMR prompts and generalized informational videos to patients [34]. Though encouraging, single‐session trainings are unlikely to have sustained impact given high staff and provider turnover reported across healthcare settings, including Title X‐funded clinics [20].

To fill this gap and make trainings, TA and resources sustainably available to Title X clinics in Atlanta, we patterned the development and deployment of our tailored bundle of capacity‐building strategies after the ISF, which includes identifying a centralized training/TA entity (i.e. the Prevention Support System). A centralized training/TA entity, such as Title X state grant holders (e.g. GFPS), allows for sustained implementation support as clinical sites can continue to request specific trainings, TA and resources that meet their unique needs over time. Our findings demonstrate that metro Atlanta Title X clinics: (1) utilized the centralized bundle of trainings, TA and resources; (2) found them acceptable; (3) tailored strategy use to their clinical groups’ needs (as evidenced by variability in requests by group); (4) felt the bundle contributed to improved HIV testing and PrEP implementation in their clinics; and (5) noted the desire for re‐training or additional training due to staff turnover. Follow‐up FGs also identified additional implementation strategies, such as audit and feedback, developed EMR prompts and workflow/process mapping, which are all within the scope of GFPS's ability to provide in support of HIV prevention services at their Title X sites.

It is notable that the trainings, TA and resources were accessible to clinical groups at any time throughout the study, and remain accessible via request still today, yet clinics, despite indicating the need for re‐training, infrequently continued their engagement with the training elements of the implementation strategy bundle. Also, in several instances when asked about what additional trainings or support could be provided by GFPS to support HIV prevention services in their clinics, responses included trainings, TA and resources that were already part of the implementation strategy bundle (e.g. insurance navigation training, PrEP implementation planning TA, follow‐in TA support for any training, TA on integrating EMR prompts). In light of notable staff and leadership turnover in Title X‐funded clinical groups, to enhance ongoing and expanded engagement with the implementation strategy bundle by clinical groups, more active and routine advertising of the bundle within the Title X clinical network may improve awareness and utilization of the variety of supportive strategies in the bundle, particularly TA, which was grossly underutilized.

The ISF also includes the Prevention Synthesis and Translation entity, which distils information about new innovations and translates it to user friendly formats as they work with the Prevention Support System to facilitate adoption of new practices like PrEP in clinical service sites. In our system, this role was held by an academic team who developed trainings and materials based on research and clinical expertise for use by GFPS with their clinical sites. This partnership is highly sustainable, as producing trainings and sharing them (e.g. train‐the‐trainer) is already a common practice for researchers and done without funding. The value of this sustained partnership is that it allows for trainings to be updated with new PrEP products as they become available, as new guidelines are published, or the development of new, adjacent trainings, TA or resources as needs arise in the clinical sites, such as those recommended in our follow‐up FGs (e.g. HIV/PrEP stigma). Thus, the ISF provides a strong model to sustainably address HIV prevention capacity‐related needs in Title X clinics in metro Atlanta, but because GFPS supports a statewide network of Title X‐funded sites (>180 across GA), the potential to scale these strategies across the state is possible as well. Importantly, given that Title X is a national programme operating in all states, the value of partnering academic and Title X agencies patterned after our use of ISF to strengthen HIV prevention practices within this healthcare system has the potential to scale nationally. Following an ISF‐guided approach is generalizable outside of the Title X network, particularly to other healthcare systems, such as the Ryan White HIV/AIDS programme [35], that have centralized training and TA support mechanisms within the system.

There are several limitations to our study. Only Title X staff from clinics located in the four EHE jurisdictions of metro Atlanta were invited to participate, so this work may not be generalizable to more rural clinical sites in GA or other areas of the United States. We also had variability across sites in the number of participants per FG, which may have resulted in missing perspectives from some clinics. We did not include patient data, only in the aggregate for HIV testing. Finally, we were not able to capture PrEP outcomes in the aggregate chart data due to logistical difficulties with the implementation of the new FPAR reporting system (FPAR 2.0), which would have included PrEP prescription data. Despite these limitations, the majority (71%) of Title X clinics in metro Atlanta participated in FGs and also received trainings, TA and/or resources on HIV testing and PrEP, so our sample does represent most of the Title X family planning network in this area. Aggregate HIV testing data for all clinical sites over 4 years is also a strength.

## 5 Conclusions

Partnership between academic institutions and Title X state‐level grantees to prepare and provide capacity‐building implementation strategies for HIV prevention is a feasible, acceptable and potentially impactful mechanism to sustainably fill gaps in HIV prevention education needs for safety net clinical networks. The ability to provide sustainable access to trainings and other HIV prevention resources via a centralized agency may be critically necessary to overcome ongoing capacity‐related barriers such as staff turnover in healthcare systems, but given this, additional strategies are needed to ensure clinics are aware of and feel comfortable accessing the implementation strategies over time.

## Author Contributions

JMS: Investigation, project administration, supervision, writing – original draft, writing – review and editing. KMA: Data curation, formal analysis, writing – original draft, writing – review and editing. DE: Investigation, project administration, supervision, writing – original draft, writing – review and editing. PW: project administration, resources, writing – review and editing. SS: Project administration, resources, writing – review and editing. MWB: Project administration, resources, writing – review and editing. MK: Project administration, supervision, writing – review and editing. AS: Investigation, project administration, supervision, writing – review and editing.

## Funding

This research is funded by the National Institute of Mental Health grant R01MH128045. This work was also supported by the Center for AIDS Research at Emory University (P30 AI050409). This work was supported by the National Institute of Allergy and Infectious Diseases.

## Conflicts of Interest

The authors have no conflicts of interest to disclose.

## Data Availability

Data are available upon reasonable request from the principal investigators, Jessica M. Sales and Anandi N. Sheth.
